# The post-genomic era of biological network alignment

**DOI:** 10.1186/s13637-015-0022-9

**Published:** 2015-06-04

**Authors:** Fazle E Faisal, Lei Meng, Joseph Crawford, Tijana Milenković

**Affiliations:** 1 Department of Computer Science and Engineering, University of Notre Dame, Notre Dame, IN, 46556 USA; 2 Interdisciplinary Center for Network Science and Applications, University of Notre Dame, Notre Dame, IN, 46556 USA; 3 ECK Institute for Global Health, University of Notre Dame, Notre Dame, IN, 46556 USA

**Keywords:** Biological network research, Protein-protein interactions, Network alignment, Across-species knowledge transfer, Functional orthology, Aging

## Abstract

Biological network alignment aims to find regions of topological and functional (dis)similarities between molecular networks of different species. Then, network alignment can guide the transfer of biological knowledge from well-studied model species to less well-studied species between conserved (aligned) network regions, thus complementing valuable insights that have already been provided by genomic sequence alignment. Here, we review computational challenges behind the network alignment problem, existing approaches for solving the problem, ways of evaluating their alignment quality, and the approaches’ biomedical applications. We discuss recent innovative efforts of improving the existing view of network alignment. We conclude with open research questions in comparative biological network research that could further our understanding of principles of life, evolution, disease, and therapeutics.

## Review

### Introduction

Bioinformatics research has revolutionized our understanding of cellular functioning. The field has opened avenues to unveil complex biological mechanisms and their connections to disease. Genomic sequence alignment, in particular, has improved our biomedical knowledge by finding sequence regions of similarities between genes in different species, where the regions likely reflect functional and evolutionary relationships between the sequences [1–4]. However, genes or their protein products do not function in isolation; rather, they carry out cellular processes by interacting with each other. This is what biological networks model, such as protein-protein interaction (PPI), gene regulatory, or metabolic networks. In a biological network, nodes represent biomolecules (such as genes or proteins), and edges represent physical or functional interactions between the biomolecules (such as PPIs). For simplicity, henceforth, we use terms “gene” and “protein” interchangeably. Unlike genomic sequence research, biological network research allows for studying complex cellular processes that emerge from the collective behavior of the biomolecules.

Due to advancements in high-throughput biotechnologies (such as yeast two-hybrid (Y2H) assays [5] or affinity purification coupled to mass spectrometry (AP/MS) [6]), large-scale PPI and other network data have become available for many species [7–16]. Given the availability of the interactome data, network research is promising to further our understanding of processes of life, evolution, and therapeutics. In particular, analogous to genomic sequence alignment, *biological network alignment* aims to find good node mapping between networks of different species that identifies topologically and functionally similar (i.e., conserved) network regions. Then, network alignment can be used to efficiently transfer the knowledge of cellular functioning from well-studied model species, such as yeast *Saccharomyces cerevisiae*, fly *Drosophila melanogaster*, or worm *Caenorhabditis elegans*, to less well-studied human, between the conserved network regions [17–19].

Biological network alignment gains importance because many proteins remain largely functionally unannotated [20–22], especially in human and other species relevant for studying disease [22, 23]. Importantly, many crucial biological processes and diseases in human are hard to study experimentally, and hence, the corresponding knowledge needs to be transferred from model species [24–30]. Human aging is an example of such a biological process. Because susceptibility to many prevalent diseases increases with age, studying human aging could aid therapeutics. Yet, human aging is hard to study experimentally due to long human lifespan as well as ethical constraints. Thus, aging-related knowledge in human needs to be obtained computationally, by transferring experimentally obtained aging-related knowledge from model species to human. Traditionally, this transfer has relied on genomic sequence alignment [31]. However, biological network data and genomic sequence data can give complementary biological insights [32–35], implying that analyses of network data can elucidate functional knowledge that cannot be extracted from sequence data by current methods. Thus, restricting alignment to sequences may limit the knowledge transfer [32–36]. For example, ∼20 % of aging-related genes in model species do not have sequence-based orthologs in human [37]. And while sequence alignment can thus not transfer this knowledge between the species, network alignment can be used to identify network-based functional orthologs across the species and thus further our knowledge of aging. Similar holds for many other biological processes and diseases.

In addition to across-species transfer of functional knowledge discussed above, just as sequence alignment, network alignment can also be used to infer phylogenetic relationships of different species based on similarities between their biological networks [38–40]. We note that in the biomedical domain, network alignment has mostly been used in the context of PPI networks. However, the problem is applicable to other types of biological networks, such as gene co-expression networks [41]. Further, network alignment has applications outside of the biomedical domain [42], with implications on, e.g., user privacy in online social networks [43].

Unlike the computationally tractable “linear” sequence alignment, exact alignment of large networks, such as biological ones, is computationally intractable due to the nondeterministic polynomial time (NP)-completeness of the underlying subgraph isomorphism problem, which asks if a network exists as an exact subgraph of another network [44]. Therefore, efficient heuristic approaches need to be sought to solve the network alignment problem approximately.

**Fig. 1:**
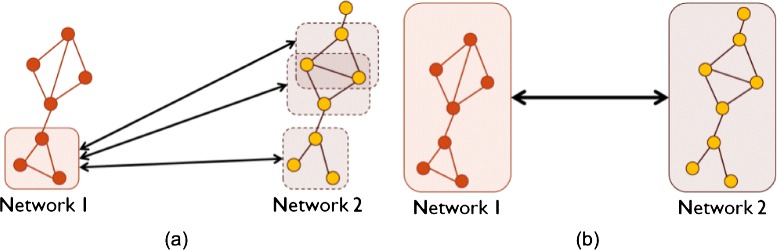
Illustration of **a** local and **b** global network alignment

Similar to sequence alignment, network alignment approaches (or network aligners) can be *local* and *global*. Local network alignment aims to find smaller network regions, such as biological pathways or protein complexes, which are highly conserved between larger input networks (Fig. 1a; for a more formal description, see the following sections). Initial network alignment efforts have focused on local alignment [45–54]. However, local aligners are generally not capable of finding *large* subgraphs that are topologically and functionally conserved between input networks. Therefore, most of the recent efforts have focused on global network alignment [18, 19, 25, 38–40, 43, 55–82], which typically aims to map well (almost) entire networks to each other. As such, global alignment is typically capable of finding large subgraphs that are conserved between input networks but at potential expense of *suboptimally* matching local network regions (Fig. 1b; for a more formal description, see the following sections).

**Fig. 2:**
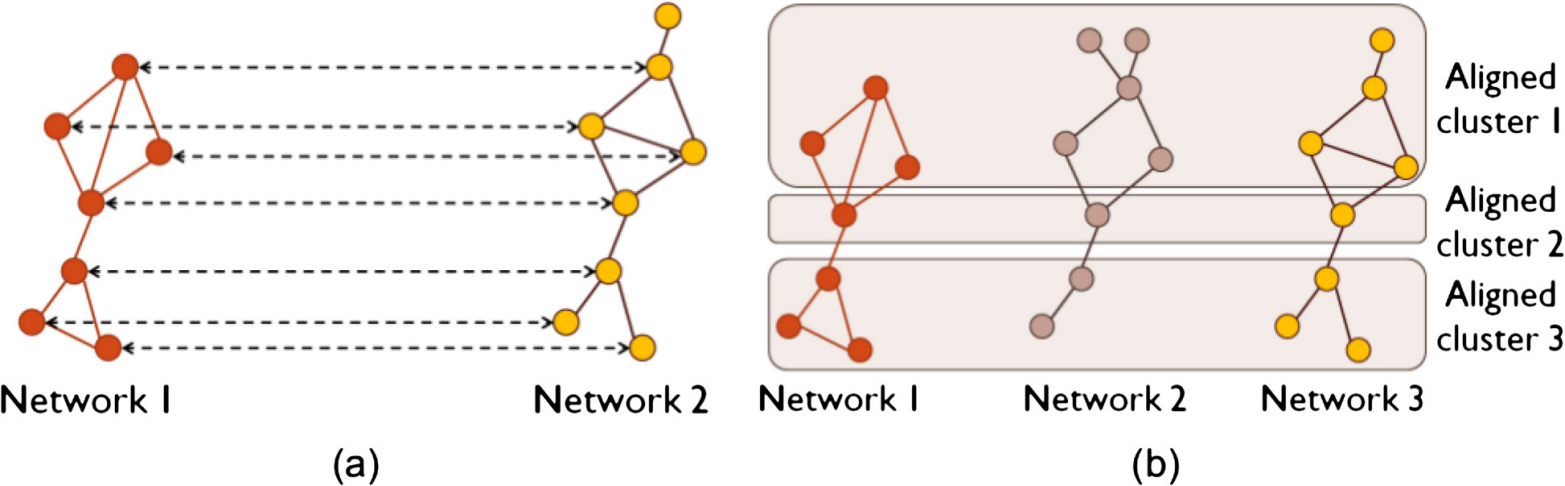
Illustration of **a** pairwise and **b** multiple network alignment

A network alignment approach can also be categorized as either *pairwise* or *multiple*, based on how many networks it can align at once. Pairwise network alignment aligns two networks at a time (Fig. 2a), whereas multiple network alignment can align more than two networks at the same time (Fig. 2b).

There exists an additional categorization of network alignment approaches into *one-to-one* or *many-to-many* methods. One-to-one network alignment produces one-to-one (or injective) node mapping, where a node from a given network can be mapped to at most one unique node from another network (Fig. 2a). On the other hand, many-to-many network alignment produces many-to-many node mapping, where a node from a given network can be mapped to several nodes from another network (Fig. 2b). We note that there also exists an approach that produces *one-to-many* node mapping, meaning that it maps a node from a given network to multiple nodes from another network, while a node from the later network can be mapped to at most one node from the former network [83].

To date, all local aligners have been of the many-to-many type, while global aligners have been of both one-to-one and many-to-many types. Further, one-to-one global aligners have traditionally been associated with pairwise alignment. In this context, nodes in the smaller of the two aligned networks are injectively mapped to nodes in the larger network, thus resulting in aligned node *pairs* (Fig. 2a). Similarly, many-to-many global aligners have traditionally been associated with multiple alignment. In this context, the output is aligned node *clusters* rather than pairs, where each cluster can contain multiple nodes from the same network (Fig. 2b). Recently, “hybrid” approaches have appeared, such as one-to-one alignment of multiple networks [75, 77, 78]. In this case, an aligned node cluster can contain at most one node from each of the aligned networks, and each node can appear in at most one aligned cluster. Table 1 categorizes some of the most prominent network alignment approaches as either local or global, pairwise or multiple, and one-to-one or many-to-many. The approaches are discussed in more detail in the following sections.

**Table 1:** Overview of prominent network alignment approaches

Network	Local or	Pairwise or	One-to-one or	Link to the
aligner	global?	multiple?	many-to-many?	software
PathBLAST [93]	Local	Pairwise	Many-to-many	http://www.pathblast.org
NetworkBLAST [46]	Local	Pairwise	Many-to-many	http://www.cs.tau.ac.il/~bnet/networkblast.htm
MaWISh [48]	Local	Pairwise	Many-to-many	http://compbio.case.edu/koyuturk/software/mawish
Graemlin 1.0 [47]	Local	Multiple	Many-to-many	http://graemlin.stanford.edu
NetworkBLAST-M [94]	Local	Multiple	Many-to-many	http://www.cs.tau.ac.il/~bnet/License-nbm.htm
NetAligner [54]	Local	Pairwise	Many-to-many	http://netaligner.irbbarcelona.org
AlignNemo [52]	Local	Pairwise	Many-to-many	http://www.sourceforge.net/p/alignnemo/home/Home
AlignMCL [53]	Local	Pairwise	Many-to-many	http://sites.google.com/site/alignmcl
IsoRank [55]	Global	Pairwise	One-to-one	http://groups.csail.mit.edu/cb/mna
IsoRankN [59]	Global	Multiple	Many-to-many	http://groups.csail.mit.edu/cb/mna
GRAAL [38]	Global	Pairwise	One-to-one	http://bio-nets.doc.ic.ac.uk/GRAAL_suppl_inf
H-GRAAL [39]	Global	Pairwise	One-to-one	http://www.nd.edu/~cone/software_data.html
MI-GRAAL [40]	Global	Pairwise	One-to-one	http://bio-nets.doc.ic.ac.uk/MI-GRAAL
GHOST [60]	Global	Pairwise	One-to-one	http://www.cs.cmu.edu/~ckingsf/software/ghost
SPINAL [67]	Global	Pairwise	One-to-one	http://code.google.com/p/spinal
SMETANA [74]	Global	Multiple	Many-to-many	http://www.ece.tamu.edu/~bjyoon/SMETANA
BEAMS [75]	Global	Multiple	Many-to-many	http://webprs.khas.edu.tr/~cesim/BEAMS.tar.gz
NetCoffee [76]	Global	Multiple	Many-to-many	http://code.google.com/p/netcoffee
FUSE [78]	Global	Multiple	One-to-one	Available upon e-mail request until formally published
NETAL [68]	Global	Pairwise	One-to-one	http://bioinf.modares.ac.ir/software/netal
GraphM [58]	Global	Pairwise	One-to-one	http://cbio.ensmp.fr/~mzaslavskiy/pwp_projects.html
NATALIE 2.0 [64]	Global	Pairwise	One-to-one	http://www.mi.fu-berlin.de/w/LiSA/Natalie
GEDEVO-M [77]	Global	Multiple	One-to-one	http://gedevo.mpi-inf.mpg.de/multiple-network-alignment
MAGNA [69]	Global	Pairwise	One-to-one	http://www.nd.edu/~cone/MAGNA
WAVE [73]	Global	Pairwise	One-to-one	Available upon e-mail request until formally published
GREAT [72]	Global	Pairwise	One-to-one	Available upon e-mail request until formally published
PINALOG [65]	Global	Pairwise	One-to-one	http://www.sbg.bio.ic.ac.uk/~pinalog

In the table, there are eight local and 19 global network aligners. Of the eight local aligners, six are pairwise and two are multiple, and all eight are many-to-many. Of the 19 global aligners, 13 are pairwise and six are multiple, and 15 are one-to-one and four are many-to-many. All global pairwise approaches are one-to-one, while global multiple approaches are either one-to-one or many-to-many

A general algorithmic idea behind network alignment approaches is to compute similarities between nodes in different networks with respect to some cost function and rapidly identify from all possible alignments a high-scoring alignment with respect to the node similarities. Many of the existing network alignment algorithms use within their node cost function biological information external to network topology, such as protein sequence similarities. However, to extract the most from each source of biological information, it would be good to know how much of new biological knowledge can be uncovered solely from topology before integrating it with other sources of biological information [39, 40, 54, 60, 69, 72, 73]. Only after methods for topological network alignment are developed that result in alignments of good topological *and* biological quality, it is beneficial to integrate them with other biological (e.g., sequence) data to further improve the quality.

We note that network alignment is one possible type of network comparison. There also exists *alignment-free* network comparison. Approaches of this type “simply” aim to quantify the similarity between different networks by comparing their overall topological properties (e.g., degree distributions or graphlet-based properties), ignoring in the process node correspondence between the networks and without aiming to identify conserved edges or subgraphs [84–88]. On the other hand, network alignment aims to find good node correspondence between networks that leads to highly similar conserved network regions. Thus, network alignment and alignment-free network comparisons have very different goals. Our focus is on network alignment.

Also, there is another type of network comparison, called *network querying*, which is more related to network alignment. Network querying typically evaluates whether a small query subnetwork (e.g., a simple path or a tree-like structure) exists as an exact subgraph of a larger target network, and if so, it identifies such a query-target subnetwork match. Prominent approaches of this type adopt a “color-coding” idea and ensure a confidence level of the resulting subnetwork match [89–92]. Unlike network querying, network alignment compares networks of arbitrary sizes, and also, it typically searches for an inexact subnetwork match between networks. Again, our focus is on network alignment.

In the following sections, we discuss the different types of existing prominent network alignment approaches. After we contrast the different approaches, we discuss existing measures that are used to evaluate alignment quality of the approaches. Next, we discuss very recent innovative directions that question the traditional view of the network alignment problem. Further, we discuss key biological applications of network alignment. Finally, we present open research questions in comparative biological network research that are expected to enhance personalized health care via improved understanding of cellular functioning, disease, and therapeutics.

### Local network alignment

#### Approaches for local network alignment

We first discuss pairwise and then multiple local network aligners.

*PathBLAST* [93] aligns two PPI networks to identify their conserved pathways. An alignment graph is first built, in which a node represents a pair of putative orthologs (one from each network), and an edge represents a conserved interaction. Highest-scoring paths are then searched for through the alignment graph with a dynamic programming approach based on the degree of protein sequence similarity and the interaction quality. In the process, gaps and mismatches are allowed to account for evolution variations and experimental errors in pathway structure.

*NetworkBLAST* [46] is an extension of PathBLAST that aims to identify not just simple linear pathways (as PathBLAST does) but also more complex network structures, e.g., dense functional modules or protein complexes. It does so by identifying high-scoring seeds in the alignment graph (which is similar to PathBLAST’s) and extending around the seeds in a greedy fashion.

*MaWISh* [48] is a pairwise local aligner modeling evolution (conservation and divergence) of protein interactions. In the alignment graph, evolutionary information is encoded into edge weights through the concepts of matches, mismatches, and duplication. MaWISh addresses network alignment as a maximum weight induced subgraph problem. Intuitively, it greedily grows a subgraph starting from a seed with maximum gain with respect to the cost function; a bad move (negative gain) is allowed in order to bypass a poor local optimum.

*NetAligner* [54] is a pairwise aligner featuring pathway-to-interactome, complex-to-interactome, and interactome-to-interactome alignments. Also, it is able to perform both inter- and intra-species alignment of networks of arbitrary topology. NetAligner constructs an initial alignment graph and searches for connected components in this graph to be used as seeds. Then, nodes from different seeds, which are disconnected in the initial alignment graph as they do not conserve edges, are now connected if they conserve indirect interactions (i.e., three-node paths) in one or both input networks. Finally, NetAligner searches again for connected components in such extended alignment graph, which are its output.

*AlignNemo* [52] is a recent pairwise local aligner capable of handling sparse network data. It first uses the concept of a *weighted* alignment graph, in which nodes represent pairs of orthologs from different species (just as in the previous methods’ alignment graphs), but edges are now weighted via a scoring strategy that accounts not only for directly conserved interactions but also for indirect interactions. So, the more paths connecting the two nodes and the more paths going through both nodes, the greater the edge weight. Then, a seed-and-extend strategy is used on the alignment graph to find *relatively* dense groups of nodes (i.e., proteins that have more interactions among themselves than with the rest of the network), which are AlignNemo’s output.

*AlignMCL* [53] is another recent pairwise local aligner that is robust to the choice of networks to be aligned. It is based on Markov clustering (MCL), a known graph clustering algorithm that simulates random walks using Markov chains iteratively. AlignMCL first builds a weighted alignment graph the same way as AlignNemo. Next, it applies MCL to this graph to identify conserved protein modules.

*Graemlin 1.0* [47] is an early multiple aligner. Based on a phylogenetic tree of species whose networks are being aligned, it uses a “progressive alignment” strategy by performing successively pairwise alignments of the closest network pairs. It first finds with a seed-and-extend strategy a pairwise alignment of the two closest species based on their phylogenetic relationship. Then, it transforms the resulting alignment together with unaligned nodes from the two networks into a new network for use in the next phase of the progressive alignment.

*NetworkBLAST-M* [94] is also a multiple aligner. It works with a novel representation of multiple networks, a layered alignment graph, in which each layer corresponds to a network and putative orthologs from different layers which are connected by inter-layer edges. NetworkBLAST-M then uses a seed-and-extend strategy to identify a high-scoring alignment from the layered alignment graph. Seeds come from a set of *k*-spines (a *k*-spine is a connected subgraph of size *k* with each node coming from a different layer) generated based on either identical topologies or underlying phylogeny. NetworkBLAST-M performs an expansion around the seed by iteratively adding to the alignment a *k*-spine that contributes the most to the current score, until no *k*-spine can be added or the alignment size exceeds the limit.

#### Alignment quality measures for local network alignment

A variety of approaches have been used to assess the quality of a local alignment, which is the set of conserved network modules. Generally, the resulting modules are compared against known protein complexes or other functional units to evaluate their overlap. Then, intuitively, the more conserved network modules there are that have large overlap with known protein complexes, the better the alignment quality. Popular biological alignment quality measures in the context of local network alignment, which quantify this notion of “network module–protein complex” overlap, are discussed below. We note that evaluation of topological alignment quality (e.g., of the amount of edges that are conserved by the alignment) is not common when it comes to local network alignment. This is because local aligners result in many-to-many node mappings, whereas edge conservation is typically defined with one-to-one node mapping in mind, and thus, it is not clear how to measure topological alignment quality of many-to-many aligners [38]. Plus, local network aligners are more biologically motivated, meaning that they are aimed at mapping protein complexes across networks, whereas global aligners are more mathematically motivated, aiming to solve a modification of the subgraph isomorphism problem.

1. *Gene ontology (GO) [* 95 *] semantic similarity.* GO semantic similarity aims to assess to what extent the mapped (i.e., conserved) network modules from different species are functionally related. First, GO semantic similarity is computed between each two proteins in the given module. This can be done in many ways, e.g., by averaging GO semantic similarities across the proteins’ associated GO term pairs [52, 53]. Then, semantic similarity of an entire module can be computed by averaging the resulting similarities over all mapped protein pairs within the module. Finally, the score of an alignment can be computed by summarizing the results over all mapped network modules, thus assigning a single GO semantic similarity score to the alignment. The higher the GO semantic similarity, the better the alignment quality. This measure was used by AlignNemo [52] and AlignMCL [53].
2. *Detection of known complexes.* Given a conserved module produced by a local aligner and a known protein complex, precision is the percentage of proteins in the conserved network module that are also present in the protein complex, recall is the percentage of proteins in the protein complex that are also in the network module, and *F*-score is the harmonic mean of precision and recall aiming to reconcile the two mutually contradicting measures [52, 54]. Then, the statistics can be summarized over all modules in the alignment. The higher the values of these measures, the better the alignment quality. These measures were used by NetAligner [54], AlignNemo [52], and AlignMCL [53].
3. *Specificity and sensitivity.* For each species, specificity is the percentage of functionally coherent conserved modules, i.e., modules that are statistically significantly enriched in a given GO term, out of all conserved modules. The statistical significance of the enrichment is typically done with respect to the hypergeometric test, corrected for multiple hypothesis testing [96]. Sensitivity is the number of distinct GO terms that are statistically significantly enriched in the given local alignment, i.e., in its conserved network modules. The higher the values of these two measures, the better the alignment quality. These measures were used by NetworkBLAST [94], NetworkBLAST-M [94], and Graemlin 1.0 [47].

#### Summary of local network aligners: which one to use?

**Fig. 3:**
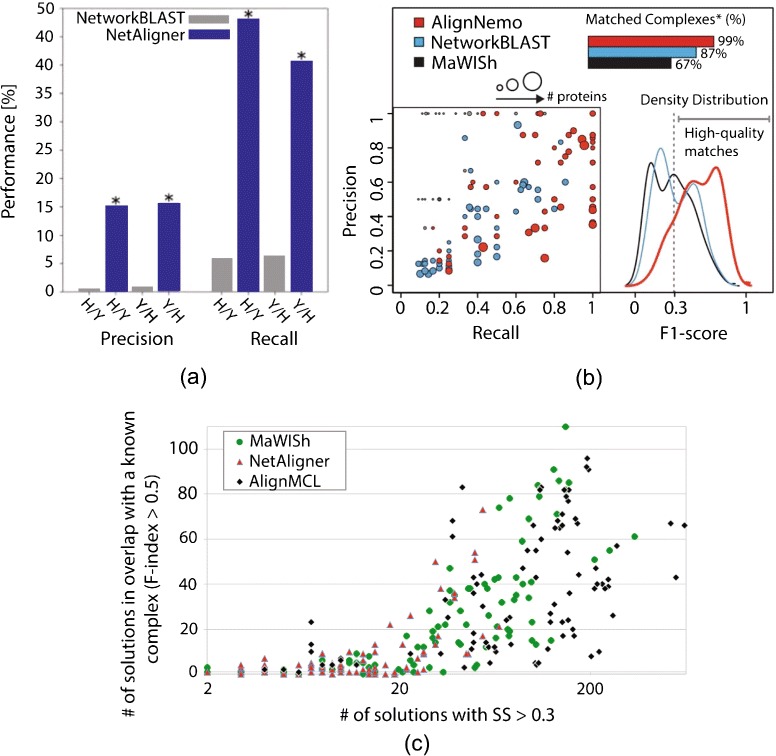
Illustration of the performance of different local network aligners. The figure compares **a** NetworkBLAST and NetAligner, **b** AlignNemo, NetworkBLAST, and MaWISh, and **c** MaWISh, NetAligner, and AlignMCL, with respect to precision, recall, or *F*-score of “aligned network module–known protein complex” matches. In **a**, precision and recall are shown when aligning networks of human (H) and yeast (Y), where the order of networks (i.e., H/Y versus Y/H) plays a role. The statistical significance of the performance difference between the two methods is indicated with an *asterisk*. In **b**, results are shown only for conserved network modules with more than six nodes, when aligning yeast and fly networks. In the lower left, precision versus recall is shown for each conserved module, represented as a *circle* whose radius is proportional to the size of the module. In the lower right, *F*-score distributions are shown for each method. Finally, at the top right, percentages are shown to quantify how well the given method’s conserved modules match known protein complexes. In **c**, each *point* represents an alignment. The position of a point on *y*-axis is determined by the number of modules (or solutions) conserved under the given alignment that match known protein complexes with *F*-score (i.e., *F*-index) above 0.5. The position of a point on *x*-axis is determined by the number of modules conserved under the given alignment that have semantic similarity (SS) scores above 0.3; semantic similarity of a conserved module quantifies functional homogeneity of proteins within the module. **a**, **b**, and **c** are adopted from [52, 54] and [53], respectively

Of the pairwise local aligners, PathBLAST [93], NetworkBLAST [46], and MaWISh [48] are among the earliest algorithms. When applied to real-world PPI networks, these pioneering aligners revealed existing as well as novel functional modules, many of which would not have been identified from sequence alignment alone. PathBLAST has been obsolete for a while now and has not been evaluated against any recent local network aligner. NetAligner was shown to be better than NetworkBLAST when identifying known functional modules in terms of precision and recall (Fig. 3a), perhaps due to NetAligner being able to handle sparse complexes, while NetworkBLAST could only identify dense complexes [54]. AlignNemo was shown to outperform both MaWISh and NetworkBLAST when detecting protein complexes, while performing comparably to NetAligner (Fig. 3b) [52]. AlignMCL was shown to generate more of high-quality conserved network modules that match known complexes well compared to NetAligner and MaWISh (Fig. 3c) [53].

Unlike the above pairwise approaches, Graemlin 1.0 [47] and NetworkBLAST-M [94] are capable of aligning multiple networks. When compared against each other, NetworkBLAST-M was shown to outperform Graemlin 1.0 in terms of specificity and sensitivity (Table 2) [94].

**Table 2:** Comparison of NetworkBLAST-M and Graemlin 1.0 on nine microbial networks in terms of specificity and sensitivity

	Specificity (%)		Sensitivity	
Species	NetworkBLAST-M	Graemlin 1.0	NetworkBLAST-M	Graemlin 1.0
*S. coelicolor*	**100.0**	71.4	**17**	12
*E. coli E12*	**90.0**	76.5	**16**	10
*M. tuberculosis*	**87.9**	76.9	**17**	8
*S. typhimurium*	**93.1**	81.3	**14**	10
*C. crescentus*	84.8	**86.7**	**15**	11
*V. cholerae*	**90.6**	80.0	**16**	9
*S. pneumoniae*	**97.0**	71.4	**14**	8
*C. jejuni*	**96.2**	76.9	**12**	9
*H. pylori*	**92.3**	56.3	**13**	8

For a given measure, the superior of the two methods is indicated in bold. The table is adopted from [94]

The above results are *overall* conclusions. Importantly, the superiority of an aligner also depends on the choice of the network data (e.g., synthetic versus real-world networks, binary Y2H versus co-complex AP/MS networks, etc.) as well as alignment quality measure and evaluation framework. Therefore, we recommend a new pairwise local aligner to be compared against AlignNemo and AlignMCL and a new multiple local aligner to be compared against NetworkBLAST-M.

### Global network alignment

#### Approaches for global network alignment

We classify prominent global network aligners into three groups according to their algorithmic design. The first group of methods employ a two-step approach: 1) use a cost function to compute pairwise similarities between nodes in different networks and 2) use an alignment strategy to rapidly identify from all possible alignments the highest-scoring alignment with respect to the total similarity over all aligned nodes [38–40, 55, 57, 59, 60, 63, 67, 74–76, 78]. Although a typical cost function aims to compute topological similarities between nodes, most of the global network aligners allow for the integration of sequence information into the node cost function. A typical alignment strategy uses the precomputed node similarity matrix resulting from the above step 1 to iteratively produce an alignment, and it does not allow for updating the matrix while creating an alignment. However, nodes that are already aligned at a given iteration of the alignment strategy might convey valuable information for guiding the remaining iterations of the strategy. Therefore, it could be desirable to update the initial node similarity matrix resulting from step 1 in each iteration of step 2. Motivated by this, the second group of recent global network aligners allows for *iteratively* updating the node similarity matrix while producing the alignment [68], or they employ an alternative similar idea [58, 64, 77]. Finally, the third group of very recent network aligners proposes a novel view of the network alignment problem (see below for details) [25, 69, 72, 73]. Next, we discuss these three approach groups.

**Two-step global network aligners** Traditional global network aligners employ the above two-step approach to produce alignments, and they mostly differ in which node cost function and alignment strategy they use, as follows.

*IsoRank* has its pairwise version [55] and its multiple version [57]. Both exploit the idea of Google’s PageRank algorithm [97] to define the same cost function. Intuitively, two nodes from different networks are similar if their network neighbors are similar, where the neighbors are similar if their own neighbors are similar, and so on. In each of the two IsoRank versions, the alignment strategy aligns in one-to-one fashion nodes between different networks greedily with respect to the cost function. IsoRank has further evolved into *IsoRankN* [59], a different multiple network aligner that uses the same cost function as IsoRank, but that uses a different, spectral graph theoretic alignment strategy to produce a *many-to-many* alignment. Intuitively, this alignment strategy is similar to *PageRank-Nibble* algorithm [98], and it finds dense clusters of nodes from multiple networks to produce aligned clusters, where each cluster can contain multiple nodes from the same network.

The *GRAAL* family of pairwise aligners [38–40], developed in parallel with the IsoRank family, uses graphlet (or small induced subgraph [84, 99]) counts to compute mathematically rigorous topological node similarity scores [100–102]. Intuitively, two nodes are a good match if their *extended* network neighborhoods are topologically similar with respect to the graphlet counts. It is the alignment strategies of the GRAAL family members that are different. The alignment strategy of the original *GRAAL* [38] is a *greedy* seed-and-extend approach, while the alignment strategy of *H-GRAAL* [39] is an *optimal* approach that aims to solve the maximum weight bipartite matching problem using Hungarian algorithm [44]. More recent *MI-GRAAL* [40] combines alignment strategies of GRAAL and H-GRAAL to further improve alignment quality. In parallel to MI-GRAAL, *C-GRAAL* [63] has appeared, whose node cost function is based on the idea of shared network neighbors rather than graphlet counts. Its alignment strategy is a seed-and-extend approach.

More recent and also pairwise *GHOST* [60] uses “spectral signatures” to compute node similarities. GHOST’s alignment strategy is similar to MI-GRAAL’s, except that MI-GRAAL solves a linear assignment problem by taking into account the similarities between nodes in different networks, while GHOST heuristically solves a quadratic assignment problem by also taking into account the similarities between nodes within the same network.

Similar to IsoRank, *SPINAL* [67] computes the similarity between two nodes based on the confidence that their neighbors can be matched well. This is done via an iterative approach that stops once a converged node similarity matrix is obtained. Given the node similarities, the aligner uses a seed-and-extend approach to produce an alignment.

The remaining aligners in this two-step category are *multiple* rather than pairwise approaches. *SMETANA* [74] bases its node cost function on a semi-Markov random walk model. Its alignment strategy uses a greedy approach to produce aligned node clusters. *BEAMS* [75] uses protein sequence similarity as its node cost function. Then, given *k* networks, its alignment strategy constructs a *k*-partite node similarity graph, identifies a set of disjoint cliques from the similarity graph that maximizes the number of conserved edges between each pair of cliques, and repeatedly merges the cliques to form aligned node clusters until the alignment score (with respect to edge conservation) can no longer be maximized. *NetCoffee’s* [76] cost function is based on the likelihood that two given proteins (from different networks) are topologically conserved. Its alignment strategy constructs a weighted bipartite graph for each pair of networks, searches for candidate edges from each bipartite graph (i.e., candidate aligned node pairs) by solving maximum weight bipartite matching problem, and finally produces an alignment over all network pairs using a simulated annealing-based approach guided by an objective function; the objective function is a measure of the summation of candidate edge weights, where the weight is defined by the node cost function. *FUSE* [78] bases its cost function on a non-negative matrix tri-factorization technique. Given *k* networks, its alignment strategy constructs a weighted *k*-partite graph and solves in approximate fashion the maximum weight *k*-partite matching problem to produce one-to-one multiple network alignment.

**Iterative global network aligners** The following approaches allow for updating node similarity scores computed with respect to the given cost function during each iteration of the alignment strategy, or they employ a similar idea of iteratively improving the alignment as it is built via an optimization strategy.

*NETAL* [68] is a pairwise aligner that iteratively recomputes the similarity between two currently unaligned nodes based on the current alignment and the expected number of conserved interactions incident to the two nodes if the nodes were to be aligned. *GraphM* [58] is a pairwise aligner that employs a gradient ascent-based iterative approach guided by an objective function to iteratively find a high-scoring alignment with respect to the objective function. GraphM uses two variations of the objective function. The first variation is based on the overall protein sequence conservation in the alignment. The second variation is based on both the sequence and edge conservation in the alignment. Motivated by the mathematical foundations of *NATALIE* [62], *NATALIE 2.0* [64] formulates the network alignment problem as a quadratic assignment problem (similar to GHOST) that is then generalized into an integer linear programming problem. Given the NP-completeness of the latter, the aligner adapts a Lagrangian relaxation approach to solve the problem using a subgradient optimization.

Unlike the above three pairwise one-to-one aligners, *GEDEVO-M* [77] is a multiple (also one-to-one) aligner. It generalizes the concept of *graph edit distance (GED)* between two networks (which is the minimum number of edge insertions and deletions needed to transform one network into another) into GED for multiple networks, in order to solve multiple network alignment problem by iteratively optimizing a GED-based objective function.

**Novel views of network alignment** Recently, two major drawbacks have been recognized with the current view of the network alignment problem, as follows.

***Mix-and-match-based network aligners.*** Recall that many of the existing global network aligners rely on the two-step algorithmic idea: node cost function and alignment strategy. Most of them have their own cost functions and alignment strategies (see above). As a result, when a network aligner is found to be superior to another, it is not clear whether this superiority comes from the first aligner’s cost function, its alignment strategy, or both. So, to fairly evaluate different two-step approaches, one should compare their different node cost functions under the same alignment strategy, for each alignment strategy, as well as their different alignment strategies under the same node cost function, for each node cost function [25, 71]. This way, one can properly evaluate which node cost function or alignment strategy is superior. Also, in the process, the combination of cost function of one method and alignment strategy of another method could outperform each original method.

**Fig. 4:**
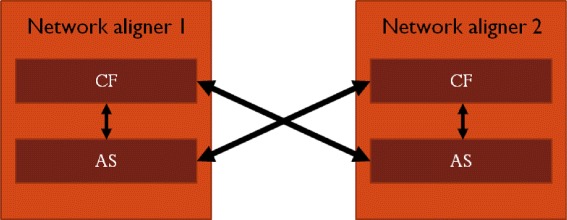
Fair evaluation of two-step global network aligners. To fairly evaluate two aligners, one should mix and match their node cost functions (CFs) and alignment strategies (ASs) and compare the different CFs under the same AS, and vice versa

Motivated by this, recent efforts have been made to mix-and-match node cost functions and alignment strategies of prominent existing network aligners, in order to perform a fair evaluation of their two algorithmic components (Fig. 4) [25, 71]. In particular, by comparing the three intuitively similar node cost functions of IsoRank family, GRAAL family, and GHOST, according to which two nodes are similar if their extended network neighborhoods are similar (see above), it was established that the GRAAL family’s graphlet-based node similarity measure is superior to those of IsoRank’s family as well as GHOST [25, 71]. On the other hand, which alignment strategy is superior depends on the choice of data set or evaluation criteria. But nonetheless, in each of the two efforts [25, 71], novel superior network aligners have been proposed that combine cost function of one method and alignment strategy of another method, confirming that it is important to properly and fairly evaluate the two-step approaches as described above.

***Edge-focused network aligners.*** Traditional network aligners discussed so far (with exception of BEAMS and GraphM) identify from possible alignments the high-scoring alignments with respect to the overall node similarity (or node conservation). However, the accuracy of the alignments is then evaluated with some other measure that is different than the node similarity used to construct the alignments. Typically, one measures the amount of conserved edges. Thus, the traditional methods align similar *nodes* between networks hoping to conserve many *edges* (*after* the alignment is constructed!).

Instead, *MAGNA* [69] has recently been proposed that directly optimizes edge conservation *while* the alignment is being constructed, without decreasing in the process the quality of node mapping. Intuitively, this aligner exploits the idea of a genetic algorithm to crossover via a novel mathematical concept two parent alignments into a superior child algorithm. MAGNA simulates the population of alignments that evolves over time for as long as allowed by computational resources and allows the fittest (highest scoring with respect to edge conservation) alignments to proceed to the next generation. Importantly, the initial population of alignments can consist of either random alignments or alignments from existing methods. Thus, MAGNA can work on top of the alignments of the existing methods to further improve their quality. But importantly, MAGNA improves upon the existing network alignment methods (that optimize node conservation rather than edge conservation) even when run on top of random alignments. MAGNA was recently extended into *MAGNA++* framework [82], in order to *simultaneously* optimize both node and edge conservation, which further improves alignment quality. Further, MAGNA++ features a user-friendly graphical interface for domain (e.g., biological) scientists while also offering source code for easy extensibility by computational scientists.

Another edge-focused alignment effort is *WAVE* [73], which is not a complete aligner *per se*, but instead, a novel iterative alignment strategy that can be used on top of any existing node cost function. Importantly, just as MAGNA++, WAVE aims to optimize both node *and* edge conservation during the alignment process, unlike previous alignment strategies. For this, it uses a novel measure of edge conservation that (unlike existing measures that treat each conserved edge the same) weighs each conserved edge so that edges with highly similar end nodes (with respect to the cost function) are favored. Using WAVE on top of established node cost functions has led to superior alignments compared to the existing methods that optimize only node conservation or only edge conservation or that treat each conserved edge the same.

In parallel to WAVE, *GREAT* [72] has appeared, which just like WAVE optimizes both node *and* edge conservation and also weighs each conserved edge to favor conserved edges that are topologically similar over conserved edges that are topologically dissimilar. Unlike WAVE, GREAT approaches the network alignment problem from a novel perspective, by aligning well *edges* between networks first in order to improve the node cost function needed to then align well nodes between the networks. GREAT, the edge-based network aligner, outperforms fairly comparable node-based network aligners. Also, it improves upon the most recent state-of-the-art methods that aim to optimize node conservation only or edge conservation only or that treat each conserved edge the same.

We note an alternative novel view of the network alignment problem. Namely, unlike the other network aligners, *PINALOG* [65] first detects clusters (dense subnetworks) in the input networks, aligns the clusters between the networks, and finally aligns nodes within the aligned clusters using a seed-and-extend approach.

#### Alignment quality measures for global network alignment

Unlike local network alignment that is typically evaluated only biologically (see above), global network aligners are evaluated both topologically and biologically. Depending on whether a global alignment is pairwise or multiple, given the difference in their input (aligned node pairs versus aligned node clusters), different measures of alignment quality are used for the different approach categories, as follows.

**Alignment quality measures for pairwise network aligners** Recall that all pairwise global aligners are also one-to-one in nature. Intuitively, a good network aligner should match well nodes between aligned networks, conserve many edges, and find a large common connected subgraph. With this in mind, the following *topological* quality measures are widely used by pairwise one-to-one aligners.

1. *Node correctness (NC).* NC of an alignment is the percentage of nodes in the smaller network (in terms of the number of nodes) that are correctly aligned (according to the ground truth node mapping) to nodes in the larger network [38]. Unlike other measures discussed below, NC is applicable only when the actual ground truth node mapping between networks is known, which is rarely the case for real-world networks. Thus, NC is typically computed on synthetic network data, when aligning a network to its noisy counterparts obtained by, e.g., randomly adding or rewiring a percentage of edges in the original network [38–40, 60, 69]. The higher the NC score, the better the alignment quality. This measure was used by GRAAL [38], H-GRAAL [39], MI-GRAAL [40], GHOST [60], NETAL [68], MAGNA [69], GREAT [72], and WAVE [73], as well as in a follow-up study on fair evaluation of existing aligners [71].
2. *Symmetric substructure score (S* ^3^ *).* *S* ^3^ measures the amount of edge conservation between two aligned networks [69]. This measure has been introduced to overcome the drawbacks of two other similar measures: *edge correctness (EC)* [38] and *induced conserved structure (ICS)* [60]. Namely, EC was proposed to measure the percentage of edges from the smaller network that are mapped to edges from the larger network under the given alignment. However, EC might fail to differentiate between two alignments that one might consider to be of different topological quality, as it is defined with respect to the smaller network but not the larger one [60]. Thus, ICS was defined as the percentage of edges from the subgraph of the larger network that participates in the alignment, which are mapped to edges in the smaller network under the given alignment [60]. However, now ICS is defined with respect to the larger network but not the smaller one. That is, since EC is defined with respect to the smaller network, it penalizes the alignment for having misaligned edges in the smaller network but not in the larger network. On the other hand, since ICS is defined with respect to the larger network, it penalizes the alignment for having misaligned edge in the larger network but not in the smaller network. With this motivation, *S* ^3^ has recently been proposed to improve upon EC and ICS by penalizing for misaligned edges in both the smaller and larger networks [69]. The higher the *S* ^3^ score, the better the alignment quality. This 2014 measure was used by MAGNA [69], GREAT [72], and WAVE [73], as well as in a follow-up study on fair evaluation of existing aligners [71]. In addition, its predecessors EC and ICS were used by GRAAL [38], H-GRAAL [39], MI-GRAAL [40], GHOST [60], and NETAL [68].
3. *Size of the largest connected common subgraph (LCCS).* Of two alignments with similar *S* ^3^ scores, one could expose large, contiguous, and topologically complex regions of network similarity, while the other could fail to do so. Thus, in addition to counting aligned edges, it is important that the aligned edges cluster together to form large connected subgraphs rather than being isolated. In this context, a connected common subgraph (CCS) is defined to be a connected subgraph (not necessarily induced) that appears in both networks [39, 40]. The size of the largest CCS (LCCS) can be measured in terms of the number of both nodes and edges [38, 40], and a new summary measure reconciling the two, which also penalizes for misaligned edges in both networks (just as *S* ^3^ does), has been proposed [69]. The higher the LCCS score, the better the alignment quality. This measure was used by IsoRank [55], GraphM [58], and most of the aligners that also used NC (see above).

The following *biological* quality measures are widely used by pairwise aligners.

1. *GO correctness* is the percentage of aligned protein pairs in which the two proteins *share* at least *k* GO terms [95], out of all aligned protein pairs in which both proteins are annotated with at least *k* GO terms [38]. GO correctness can be computed with respect to complete GO annotation data, independent of GO evidence code. However, since many GO annotations have been obtained via sequence comparison, and since some of the aligners also use sequence information to produce their alignments, GO correctness can be biased by the sequence information. Therefore, it is highly recommended to consider only GO annotations with experimental evidence codes when computing GO correctness of an aligner that uses sequence information [38]. This measure was used by GRAAL [38], H-GRAAL [39], MI-GRAAL [40], C-GRAAL [63], GHOST [60], NETAL [68], MAGNA [69], and WAVE [73], as well as in a follow-up study on fair evaluation of existing aligners [71].
2. *GO semantic similarity.* We have already discussed this measure above in the context of local network alignment. It complements GO correctness, as follows. GO correctness is a stricter measure that requires two proteins in an alignment to share one or more common GO terms. However, two proteins can still be functionally similar if they share a similar GO term, without necessarily sharing the same GO term. GO correctness would fail to identify such functional similarity between two proteins. GO semantic similarity between two proteins overcomes the limitation by taking into account the semantic similarity between GO terms that the two proteins are annotated with [103–107]. Just as with local aligners, GO semantic similarity between two proteins can be computed in many ways, e.g., by averaging GO semantic similarities across the proteins’ associated GO term pairs [52, 69, 108]. Then, GO semantic similarity of an entire alignment can be computed by averaging the resulting similarities over all protein pairs in the alignment, thus assigning a single semantic similarity score to the alignment [52, 69, 108]. This measure was used by GHOST [60] and MAGNA [69].

**Alignment quality measures for multiple network aligners** Recall that most of multiple network aligners are also many-to-many in nature, meaning that multiple nodes in one network can be mapped to multiple nodes in another network, resulting in a set of aligned node clusters. The clusters are typically non-overlapping, but a cluster can contain multiple nodes from the same network. For these reasons, different alignment quality measures are required for multiple network aligners compared to pairwise aligners [59]. Intuitively, a good multiple network aligner should produce aligned clusters such that nodes in each cluster are functionally uniform or *consistent*. Also, it should produce many such clusters, so that it *covers* as many of the nodes from the aligned networks as possible. The following alignment quality measures are widely used for a multiple network aligner. Among them, the first one is a topological measure and the remaining ones are biological measures. (Note that an additional potential measure of topological alignment quality exists, as follows. BEAMS generalizes the concept of edge conservation from pairwise to multiple alignment [75]. Whereas BEAMS uses this measure to define its alignment score that is optimized while creating a multiple alignment, nothing prevents one to use this measure to evaluate topological quality of another aligner’s multiple alignment after it is created.)

1. The larger *the number of aligned clusters* containing at least three nodes, the better the alignment quality. This measure was used by IsoRankN [59], as well as in a follow-up study on fair evaluation of existing aligners [25].
2. A related measure is *k-coverage*, which counts the number of clusters containing proteins from *k* different networks. This measure was used by SMETANA [74], BEAMS [75], and FUSE [78].
3. *Exact cluster ratio* is the percentage of aligned clusters in which all proteins share a GO term. The higher its value, the better the alignment quality. This measure was used by IsoRankN [59] and in a follow-up study on fair evaluation of existing aligners [25].
4. *Exact protein ratio* is the percentage of all proteins that are in the exact clusters (as defined above). The higher its value, the better the alignment quality. This measure was used by IsoRankN [59] and in a follow-up study on fair evaluation of existing aligners [25].
5. *Mean entropy of alignment.* First, the entropy of an aligned cluster $S_{v}^{*}$ is computed as: $H(S_{v}^{*}) = H(p_{1},p_{2},\ldots,p_{d}) = -\sum _{i=1}^{d}p_{i}\log {p_{i}}$, where *p*_*i*_ is the percentage of all proteins in $S_{v}^{*}$ that have GO term *i*, and *d* is the total number of GO terms [59]. Then, the mean entropy of the alignment is obtained by averaging entropies across all clusters in the alignment. The lower the entropy of the alignment, the higher its average within-cluster GO term consistency, and consequently, the better its biological quality. This measure was used by IsoRankN [59], SMETANA [74], BEAMS [75], NetCoffee [76], GEDEVO-M [77], and FUSE [78], as well as in a follow-up study on fair evaluation of existing aligners [25].
6. *Normalized mean entropy of alignment*. First, the normalized entropy of an aligned cluster $S_{v}^{*}$ is computed as: $\bar {H}(S_{v}^{*}) = \frac {1}{\log {d}}H(S_{v}^{*}).$ Then, the mean normalized entropy is obtained by averaging normalized entropies across all aligned clusters. The lower the normalized mean entropy, the better its biological quality. This measure was used by IsoRankN [59], SMETANA [74], BEAMS [75], NetCoffee [76], GEDEVO-M [77], and FUSE [78], as well as in a follow-up study on fair evaluation of existing aligners [25].

**Evaluating statistical significance of an alignment** When aligning networks with an approach, it is important to measure the statistical significance of the given alignment quality score. There are several approaches to achieve this. One could compute the probability of obtaining the same or better score by aligning the actual networks with a random aligner [39, 48]. Additionally, one could compute the probability of obtaining the same or better score by aligning random networks with the actual approach [39]. In this context, random networks should come from an appropriate network null model (i.e., graph family), and many network null models exist [109, 110].

#### Summary of global network aligners: which one to use?

**Fig. 5:**
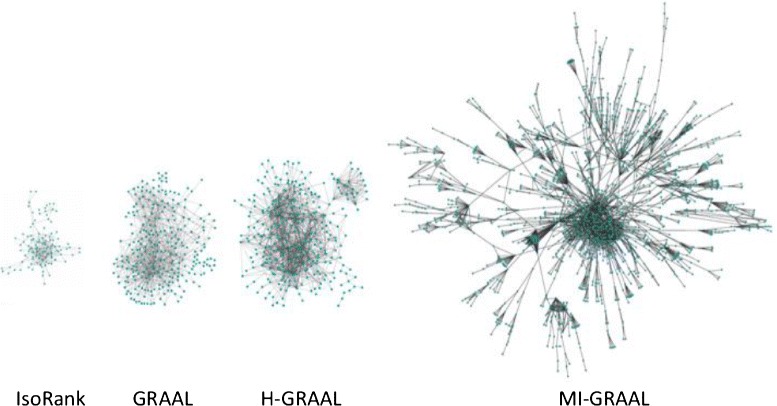
Illustration of the performance of different global network aligners with respect to LCCS. The figure shows LCCSs between yeast and human PPI networks revealed by IsoRank (116 proteins and 261 interactions), GRAAL (267 proteins and 900 interactions), H-GRAAL (317 proteins and 1290 interactions), and MI-GRAAL (1858 proteins and 3467 interactions). GHOST and MAGNA, as more recent aligners, produced even larger LCCSs (figures not shown). Namely, GHOST’s LCCS, as reported in [69], has 1622 proteins and 5356 interactions. MAGNA, when run on MI-GRAAL’s initial population, returns LCCS with 1660 proteins and 3401 interactions. MAGNA, when run on GHOST’s initial population, returns LCCS with 1327 proteins and 4363 interactions. Note that it is not the raw node and edge counts alone that should be compared between two methods’ LCCSs. Namely, a good method should find an LCCS that has many edges as well as nodes, while at the same time penalizing for edges from both the smaller and larger networks that are misaligned under the given alignment (just as *S* ^3^ does). A summary LCCS measure capturing all of this was recently proposed [69]. The resulting summary LCCS scores are 51, 54, 59, and 59 % for MI-GRAAL, GHOST, and the two MAGNA versions, respectively. Clearly, GHOST is superior to MI-GRAAL, while MAGNA is superior to GHOST (and thus to MI-GRAAL). The LCCS figures of IsoRank, GRAAL, H-GRAAL, and MI-GRAAL are adopted from the original publications [38–40]

**Fig. 6:**
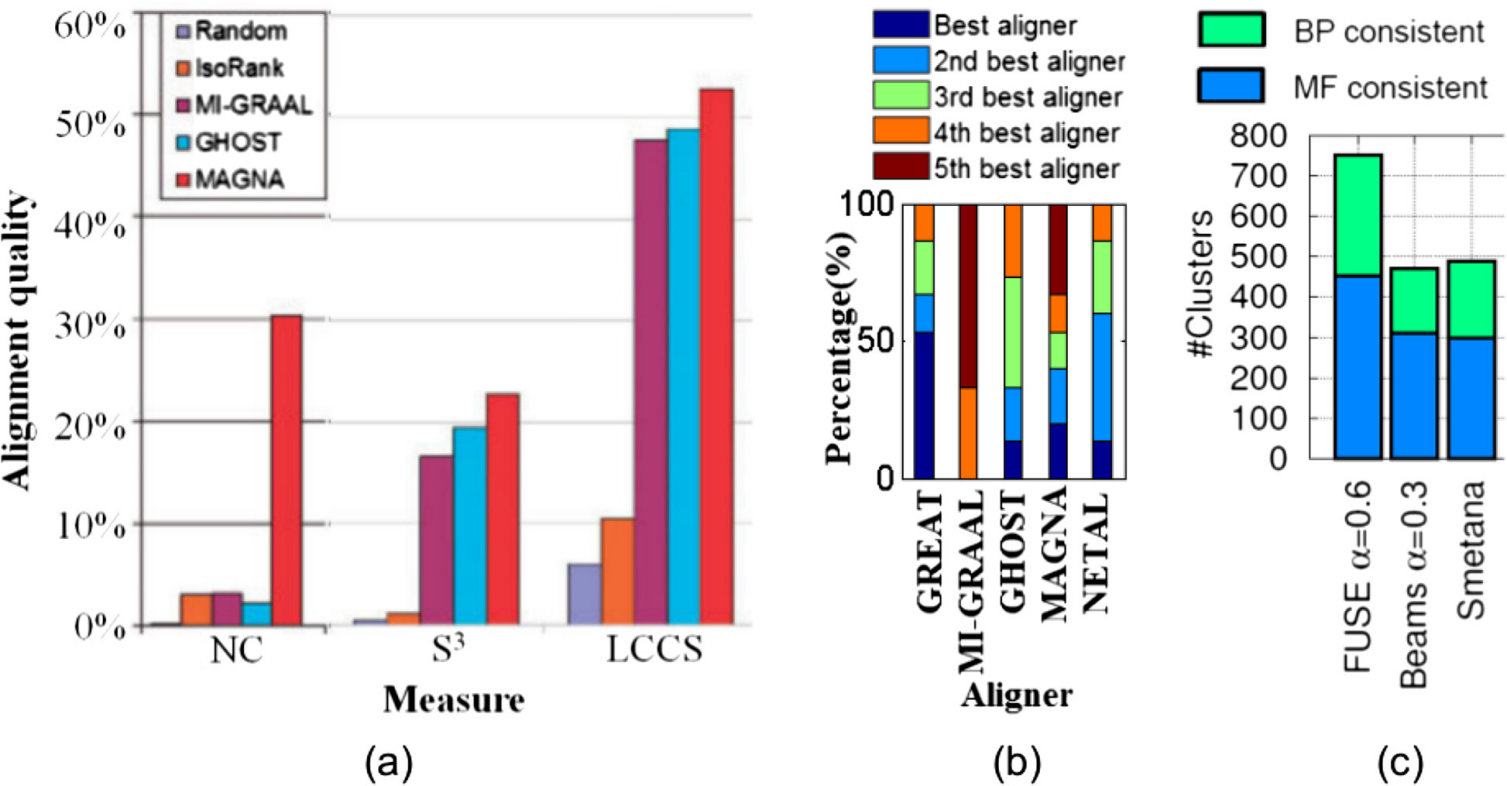
Additional illustration of the performance of different global network aligners. This figure shows **a** the superiority of MAGNA over IsoRank, MI-GRAAL, and GHOST with respect to NC when aligning synthetic noisy yeast networks with known node mapping, and with respect to *S* ^3^ and LCCS when aligning *Campylobacter jejuni* and *Escherichia coli* bacterial PPI networks [69], **b** the ranking of GREAT, MI-GRAAL, GHOST, MAGNA, and NETAL over all alignments produced by the original GREAT study [72] with respect to three alignment quality measures (NC, *S* ^3^, and LCCS) combined, demonstrating the superiority of GREAT over the other aligners [72], and **c** the superiority of FUSE (its best parameter version, as reported in [78]) over BEAMS (its best parameter version, as reported in [78]) and SMETANA with respect to the number of functionally consistent aligned node clusters, i.e., clusters that are enriched in a biological process (BP) or molecular function (MF) GO term [78]. The figures in **a**, **b**, and **c** are adopted from [69, 72] and [78], respectively

Of the pairwise global aligners, IsoRank [55], GraphM [58], GRAAL [38], and H-GRAAL [39] are among the earliest global alignment algorithms and have by now been outperformed by the newer approaches. MI-GRAAL was shown to perform significantly better than IsoRank, GRAAL, and H-GRAAL [40] (Fig. 5). MI-GRAAL and GHOST are mostly comparable to each other [71], with slight superiority of GHOST in some contexts. NETAL was shown to be either superior or comparable to MI-GRAAL [68]. MAGNA, a recent edge-based aligner, was shown to be superior to IsoRank, MI-GRAAL, and GHOST [69] (Fig. 6a). Newer MAGNA++ was shown to outperform MAGNA [82]. GREAT and WAVE, also very recent edge-focused pairwise aligners, have been shown to perform better than MI-GRAAL, GHOST, MAGNA, and NETAL [72] (Fig. 6b). The above results are *overall* conclusions. Importantly, the superiority of an aligner also depends on the choice of the network data (e.g., synthetic versus real-world networks, binary Y2H versus co-complex AP/MS networks, etc.) as well as alignment quality measure and evaluation framework. Therefore, we recommend a new pairwise global aligner to be compared against MI-GRAAL, GHOST, NETAL, MAGNA++, GREAT, and WAVE.

Of the multiple global aligners, SMETANA, BEAMS, and NetCoffee were shown to be superior to IsoRankN [74–76], while SMETANA, BEAMS, and NetCoffee are mostly comparable to each other [75, 76]. FUSE, a recent multiple aligner, has been shown to outperform SMETANA and BEAMS with respect to most of the alignment quality measures [78] (Fig. 6c). Since again, the superiority of an aligner depends on the choice of data and alignment quality measure, we recommend a new multiple global aligner to be compared against SMETANA, BEAMS, NetCoffee, and FUSE.

### Key biological implications of network alignment

Due to recent popularity of global network alignment (Table 1), our discussion in this section focuses primarily on biological results of global aligners.

#### Revealing conserved PPI network regions between yeast and human

Network aligners have been used to reveal conserved and unexpectedly large PPI network regions between different species, with special focus on yeast and human, as the two most complete PPI eukaryotic networks to date [111]. Figure 5 illustrates the size of LCCS between yeast and human PPI networks obtained by IsoRank, GRAAL, H-GRAAL, and MI-GRAAL. GHOST and MAGNA, being the more recent aligners, revealed even larger LCCSs [60, 69].

**Fig. 7:**
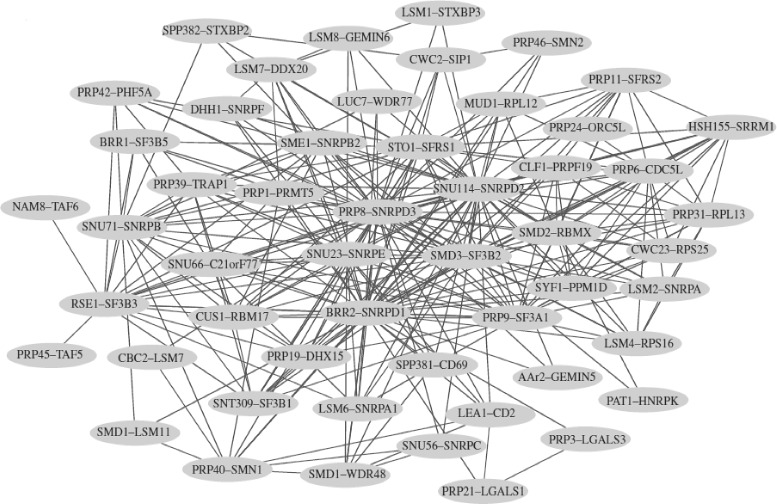
GRAAL’s second largest CCS. The second largest CCS uncovered by GRAAL when aligning PPI networks of yeast and human, consisting of 286 interactions amongst 52 proteins; each node in the CCS contains a label denoting a pair of yeast and human proteins that are aligned and each edge between two nodes means that an interaction exists in both species between the corresponding protein pairs [38]

Importantly, conserved network regions uncovered by the aligners are typically enriched in the same biological function (this is true for both local and global aligners). For example, GRAAL has aligned a subnetwork consisting of 52 nodes between yeast and human, where 98 % yeast proteins and 67 % human proteins are involved in splicing (Fig. 7) [38]. This is highly encouraging because splicing is known to be conserved among eukaryotic species, even if they are as diverse as yeast and human [112].

#### Revealing evolutionary relationships between species

Several prominent network aligners aimed to uncover evolutionary relationships between species and construct the species’ phylogenetic tree based on similarities between their biological networks. GRAAL and H-GRAAL were first such aligners. Since PPI network structure has subtle effects on the evolution of proteins and reasonable phylogenetic inference can only be done between closely related species [113], and since no PPI data were available for closely related species around the time GRAAL and H-GRAAL appeared, these methods focused on phylogenetic tree inference based on *metabolic* networks [114]. In particular, these aligners used metabolic data from KEGG [15] to reconstruct phylogenetic relationships for seven closely related organisms from the family of protist, as well as for six closely related organisms from the family of fungi [38, 39]. It was encouraging that the resulting phylogenetic trees were non-random and very similar to “ground truth” trees found by sequence comparison. The fact that the network-based trees slightly differed from those based on sequence data is not alarming, as there is no reason to believe that the sequence-based ones should a priori be considered the correct ones. This is because sequence-based phylogenetic tree inference suffers from several problems underlying sequence comparison [38, 39], which is a *computational* way of obtaining the trees, just as network alignment is.

**Fig. 8:**
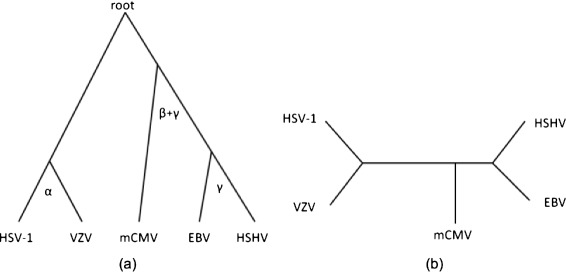
MI-GRAAL’s application to inferring phylogenetic relationships between species. Phylogenetic tree of five hyperviruses, namely varicella-zoster virus (VZV), Kaposis sarcoma-associated herpes virus (KSHV), herpes simplex virus 1 (HSV-1), murine cytomegalovirus (mCMV), and Epstein–Barr virus (EBV) [115], according to **a** the gold standard [117, 118] and **b** MI-GRAAL alignments of the species’ PPI networks [40]

Around the time MI-GRAAL appeared, genome-wide *PPI networks* became available for five closely related species from the family of herpesviruses [115]. By “genome-wide,” we mean that all possible protein pairs in each virus were tested for interactions. Thus, MI-GRAAL used these PPI network data to infer evolutionary relationships between the five herpesviruses based on their network similarities. Importantly, MI-GRAAL correctly reconstructed the phylogenetic tree (Fig. 8).

All of these results support the original hypothesis of GRAAL, H-GRAAL, and MI-GRAAL studies that biological network data is a valuable source of biological and evolutionary information.

#### Revealing new knowledge about human aging

Since the US population is on average growing older because of ∼78 million of baby boomers who began turning 65 in 2011, and since susceptibility to diseases increases with age, studying molecular mechanisms behind aging and aging-associated diseases gains importance. However, human aging is hard to study experimentally because of long lifespan and ethical constraints. Therefore, learning knowledge about human aging needs to rely on computational research. Genomic sequence alignment, a popular computational direction, has typically been used to learn about human aging by transferring the knowledge from highly studied model species to poorly studied human between conserved sequence regions. According to GenAge, one of the most trusted data sources on aging, 298 human genes are involved in the aging processes or diseases, and most of this knowledge are predictions obtained via genomic sequence comparison [116]. However, non-sequence data and genomic sequence data can give complementary biological insights. Therefore, PPI network data can be studied to elucidate aging-related knowledge missed by the current sequence-based approaches. Also, since not all genes implicated in aging in model species have sequence-based orthologs in human, restricting comparison to sequence data may limit the knowledge transfer.

Motivated by this, it was recently hypothesized that network alignment can be used to transfer the knowledge about aging from one species to another between conserved (aligned) PPI networks [25]. Indeed, it was shown that state-of-the-art network aligners at the time, MI-GRAAL and IsoRankN, as well as their mix-and-match combination, can uncover *existing* aging-related knowledge with statistically significantly high accuracy, in the sense that the methods align well known aging-related network parts of one species to known aging-related network parts of other species. Then, from the alignments, *novel* aging-related knowledge was predicted in currently unannotated network regions whenever such regions were aligned to known aging-related network regions. In this way, compared to 298 human aging-related genes from GenAge, additional 792 human genes were predicted as novel aging-related candidates [25]. These predictions were validated by demonstrating their topological and biological similarities to known aging-related genes, as well as via literature search. For example, they were found to be involved in aging-related biological processes and diseases, such as brain tumor, cancer, or prostate cancer.

We note that additional methods for network-based research of human aging exist, which are not aimed at across-species network comparison. For example, recently, the current static PPI network of human was integrated with aging-related gene expression data to construct dynamic, age-specific networks [24]. Then, genes whose network positions significantly changed with age were predicted as aging-related, and they were validated in similar ways as above. The value of this dynamic network approach is that it overcomes the key drawback of traditional biological network research, which deals with static network representations of the cellular functioning that changes with time (or age).

## Conclusions

### Future directions and concluding remarks

Comparative biological network research has attracted significant attention in the computational biology community. Nonetheless, despite all of the valuable existing efforts, many research questions remain to be addressed. For example, different network aligners, and even different parameter versions of the same aligner, tend to identify very different solutions. A struggle for a computational scientist is the current lack of in-depth understanding of the qualitative (rather than just quantitative) effect of method or parameter choice on the resulting output. A struggle for a biological scientist is which of the different alignment solutions to focus on for their experimental validation.

Moreover, despite the increasing availability of biological network data, the data remains noisy and incomplete, even for well-studied species. The effect of noise on the data on the resulting alignment(s) is poorly understood. On a related note, many different types of biological network data exist that capture somewhat complementary functional slices of the cell, whereas the network alignment community has focused their attention mainly on PPI networks. By developing efficient approaches for data integration as well as for alignment of the resulting heterogeneous network data, one could not only buffer noise in each individual network type but also uncover novel biological knowledge that would be missed by studying each individual data type in isolation.

Further, as we have discussed, many different types of network aligners exist that typically aim to achieve different goals and thus require different evaluation frameworks, which makes it hard to fairly compare the different methods. Perhaps future focus should shift towards development of “hybrid” approaches that inherit the best from all worlds while offering consistency in terms of method evaluation and comparison.

Also, regarding method evaluation, whereas accuracy is important, so is computational complexity. Thus, improvements in this aspect are needed to make the existing and future methods scalable to biological network data that will only continue to grow in size. To further ensure practical usefulness of a method, proper documentation of the software implementing the method, reliability of the software, and availability of a friendly graphical user interface are all critical for the method to be widely adopted, especially by biomedical domain scientists.

Given the tremendous amounts of biological network data that are being produced, network alignment will only continue to gain importance. Further advances in this research area could lead to new discoveries about the principles of life, evolution, disease, and therapeutics, and in the long run, they could facilitate advances in health care and personalized medicine.

## References

[1] Altschul SF, Gish W, Miller W, Lipman DJ (1990). Basic local alignment search tool. J. Mol. Biol..

[2] Biesecker LG, Mullikin JC, Facio FM, Turner C, Cherukuri PF, Blakesley RW, Bouffard GG, Chines PS, Cruz P, Hansen NF, Teer JK, Maskeri B, Young AC, Program NCS, Manolio TA, Wilson AF, Finkel T, Hwang P, Arai A, Remaley AT, Sachdev V, Shamburek R, Cannon RO, Green ED (2009). The ClinSeq project: piloting large-scale genome sequencing for research in genomic medicine. Genome Res..

[3] Tsai SQ, Iafrate AJ, Joung JK (2014). Genome editing: a tool for research and therapy: towards a functional understanding of variants for molecular diagnostics using genome editing. Nat. Med..

[4] Alföldi J, Lindblad-Toh K (2013). Comparative genomics as a tool to understand evolution and disease. Genome Res..

[5] Yu H, Braun P, Yildirim MA, Lemmens I, Venkatesan K, Sahalie J, Hirozane-Kishikawa T, Gebreab F, Li N, Simonis N, Hao T, Rual JF, Dricot A, Vazquez A, Murray RR, Simon C, Tardivo L, Tam S, Svrzikapa N, Fan C, Smet de AS, Motyl A, Hudson ME, Park J, Xin X, Cusick ME, Moore T, Boone C, Snyder M, Roth FP (2008). High-quality binary protein interaction map of the yeast interactome networks. Science.

[6] Gstaiger M, Aebersold R (2009). Applying mass spectrometry-based proteomics to genetics, genomics and network biology. Nat. Rev. Genet..

[7] Breitkreutz BJ, Stark C, Reguly T, Boucher L, Breitkreutz A, Livstone M, Oughtred R, Lackner DH, Bahler J, Wood V, Dolinski K, Tyers M (2008). The BioGRID Interaction Database: 2008 update. Nucleic Acids Res..

[8] Peri S, Navarro JD, Kristiansen TZ, Amanchy R, Surendranath V, Muthusamy B, Gandhi TK, Chandrika KN, Deshpande N, Suresh S (2004). Human protein reference database as a discovery resource for proteomics. Nucleic Acids Res..

[9] Cherry JM, Adler C, Ball C, Chervitz SA, Dwight SS, Hester ET, Jia Y, Juvik G, Roe T, Schroeder M, Weng S, Botstein D (1998). SGD: saccharomyces genome database. Nucleic Acids Res..

[10] Xenarios I, Rice DW, Salwinski L, Baron MK, Marcotte EM, Eisenberg D (2000). DIP: the database of interacting proteins. Nucleic Acids Res..

[11] Xenarios I, Salwinski L, Duan XJ, Higney P, Kim SM, Eisenberg D (2002). DIP, the Database of interacting proteins: a research tool for studying cellular networks of protein interactions. Nucleic Acids Res..

[12] Hermjakob H, Montecchi-Palazzi L, Lewington C, Mudali S, Kerrien S, Orchard S, Vingron M, Roechert B, Roepstorff P, Valencia A, Margalit H, Armstrong J, Bairoch A, Cesareni G, Sherman D, Apweiler R (2004). IntAct: an open source molecular interaction database. Nucleic Acids Res..

[13] KR Brown, I Jurisica, Unequal evolutionary conservation of human protein interactions in interologous networks. Genome Biol. 8(5), 95 (2007). 10.1186/gb-2007-8-5-r95 PMC1929159 17535438

[14] Brown KR, Jurisica I (2005). Online predicted human interaction database. Bioinformatics.

[15] Kanehisa M, Goto S, Sato Y, Kawashima M, Furumichi M, Tanabe M (2014). Data, information, knowledge and principle: back to metabolism in KEGG. Nucleic Acids Res..

[16] Kanehisa M, Goto S (2000). KEGG: Kyoto Encyclopedia of Genes and Genomes. Nucleic Acids Res..

[17] Sharan R, Ideker T (2006). Modeling cellular machinery through biological network comparison. Nat. Biotechnol..

[18] Clark C, Kalita J (2014). A comparison of algorithms for the pairwise alignment of biological networks. Bioinformatics.

[19] Malod-Dognin N, Pržulj N (2014). GR-Align: fast and flexible alignment of protein 3D structures using graphlet degree similarity. Bioinformatics.

[20] Sharan R, Ulitsky I, Shamir R (2007). Network-based prediction of protein function. Mol. Reprod. Dev..

[21] Dwight SS, Harris MA, Dolinski K, Ball CA, Binkley G, Christie KR, Fisk DG, Issel-Tarver L, Schroeder M, Sherlok G, Sethuraman A, Weng S, Botstein D, Cherry JM (2002). Saccharomyces genome database (SGD) provides secondary gene annotation using the gene ontology (GO). Nucleic Acids Res..

[22] Mulder NJ, Akinola RO, Mazandu GK, Rapanoel H (2014). Using biological networks to improve our understanding of infectious diseases. Comput. Struct. Biotechnol. J..

[23] K Sun, JP Gonçalves, C Larminie, N Pržulj, Predicting disease associations via biological network analysis. BMC Bioinformatics. 15, 304 (2014). 10.1186/1471-2105-15-304 PMC4174675 25228247

[24] Faisal FE, Milenković T (2014). Dynamic networks reveal key players in aging. Bioinformatics.

[25] Faisal FE, Zhao H, Milenkovic T (2015). Global network alignment in the context of aging. IEEE/ACM Trans. Comput. Biol. Bioinform..

[26] Magalhães de JP, Foyer CH, Faragher R, Thornalley PJ (2009). Aging research in the post-genome era: new technologies for an old problem. Redox Metabolism and Longevity Relationships in Animals and Plants.

[27] Ferrarini LA, Bertelli L, Feala J, McCulloch AD, Paternostro G (2005). A more efficient search strategy for aging genes based on connectivity. Bioinformatics.

[28] Promislow DEL (2004). Protein networks, pleiotropy and the evolution of senescence. Proc. R Soc. B: Biol. Sci..

[29] Kriete A, Lechner M, Clearfield D, Bohmann D (2011). Computational systems biology of aging. Wiley Interdiscip. Rev. Syst. Biol. Med..

[30] R Reja, AJ Venkatakrishnan, J Lee, BC Kim, JW Ryu, S Gong, J Bhak, D Park, MitoInteractome: Mitochondrial protein interactome database, and its application in ‘aging network’ analysis.BMC Genomics. 10(Suppl 3), 20 (2009). 10.1186/1471-2164-10-S3-S20 PMC2788373 19958484

[31] Altschul SF, Madden TL, Schffer AA, Zhang J, Zhang Z, Miller W, Lipman DJ (1997). Gapped BLAST and PSI-BLAST: a new generation of protein database search programs. Nucleic Acids Res..

[32] V Memišević, T Milenković, N Pržulj, Complementarity of network and sequence information in homologous proteins. J. Integr. Bioinform. 7(3), 135 (2010). 10.2390/biecoll-jib-2010-135 20375452

[33] T Przytycka, YA Kim, Network integration meets network dynamics. BMC Biology. 8(1), 48 (2010). 10.1186/1741-7007-8-48 PMC2861031 20513250

[34] Berger B, Peng J, Singh M (2013). Computational solutions for omics data,. Nat. Rev. Genet..

[35] Ryan CJ, Cimermancic P, Szpiech ZA, Sali A, Hernandez RD, Krogan NJ (2013). High-resolution network biology: connecting sequence with function. Nat. Rev. Genet..

[36] Gautheret D, Major F, Cedergren R (1990). Pattern searching/alignment with RNA primary and secondary structures: an effective descriptor for tRNA. Comput. Appl. Biosci: CABIOS.

[37] Tacutu R, Budovsky A, Fraifeld VE (2010). The NetAge database: a compendium of networks for longevity, age-related diseases and associated processes. Biogerontology.

[38] Kuchaiev O, Milenković T, Memišević V, Hayes W, Pržulj N (2010). Topological network alignment uncovers biological function and phylogeny. J. R. Soc. Interface.

[39] Milenković T, Ng WL, Hayes W, Pržulj N (2010). Optimal network alignment with graphlet degree vectors. Cancer Inform..

[40] Kuchaiev O, Pržulj N (2011). Integrative network alignment reveals large regions of global network similarity in yeast and human. Bioinformatics.

[41] Ficklin SP, Feltus FA (2011). Gene coexpression network alignment and conservation of gene modules between two grass species: maize and rice. Plant Physiol..

[42] Tang J, Lou T, Kleinberg J (2012). Inferring social ties across heterogenous networks. Proceedings of the Fifth ACM International Conference on Web Search and Data Mining.

[43] Narayanan A, Shi E, Rubinstein BIP (2011). Link prediction by de-anonymization: how we won the Kaggle social network challenge. Proceedings of the 2011 International Joint Conference on Neural Networks (IJCNN).

[44] West DB (2001). Introduction to Graph Theory.

[45] Kelley BP, Bingbing Y, Lewitter F, Sharan R, Stockwell BR, Ideker T (2004). PathBLAST: a tool for alignment of protein interaction networks. Nucleic Acids Res..

[46] Sharan R, Suthram S, Kelley RM, Kuhn T, McCuine S, Uetz P, Sittler T, Karp RM, Ideker T (2005). Conserved patterns of protein interaction in multiple species. Proc. Natl. Acad. Sci. U S A.

[47] Flannick J, Novak A, Balaji SS, Harley HM, Batzglou S (2006). Graemlin general and robust alignment of multiple large interaction networks. Genome Res..

[48] Koyutürk M, Kim Y, Topkara U, Subramaniam S, Szpankowski W, Grama A (2006). Pairwise alignment of protein interaction networks. J. Comput. Biol..

[49] Berg J, Lassig M (2004). Local graph alignment and motif search in biological networks. Proc. Natl. Acad. Sci. U. S. A..

[50] Liang Z, Xu M, Teng M, Niu L (2006). NetAlign: a web-based tool for comparison of protein interaction networks. Bioinformatics.

[51] Berg J, Lassig M (2006). Cross-species analysis of biological networks by Bayesian alignment. Proc. Natl. Acad. Sci..

[52] G Ciriello, M Mina, PH Guzzi, M Cannataro, C Guerra, AlignNemo: a local network alignment method to integrate homology and topology. PLOS ONE. 7(6), 38107 (2012). 10.1371/journal.pone.0038107 PMC3373574 22719866

[53] Mina M, Guzzi PH (2012). AlignMCL: comparative analysis of protein interaction networks through markov clustering. Proceedings of the 2012 IEEE International Conference on Bioinformatics and Biomedicine Workshops.

[54] RA Pache, P Aloy, A novel framework for the comparative analysis of biological networks. PLOS ONE. 7(2), 31220 (2012). 10.1371/journal.pone.0031220 PMC3283617 22363585

[55] Singh R, Xu J, Berger B (2007). Pairwise global alignment of protein interaction networks by matching neighborhood topology. Proceedings of the 11th Annual International Conference on Research in Computational Molecular Biology.

[56] Flannick J, Novak AF, Do CB, Srinivasan BS, Batzoglou S (2008). Automatic parameter learning for multiple network alignment. Proceedings of the 12th Annual International Conference on Research in Computational Molecular Biology.

[57] Singh R, Xu J, Berger B (2008). Global alignment of multiple protein interaction networks. Proc. Pac. Symp. Biocomput..

[58] Zaslavskiy M, Bach F, Vert JP (2009). Global alignment of protein-protein interaction networks by graph matching methods. Bioinformatics.

[59] Liao C, Lu K, Baym M, Singh R, Berger B (2009). IsoRankN: spectral methods for global alignment of multiple protein networks. Bioinformatics.

[60] Patro R, Kingsford C (2012). Global network alignment using multiscale spectral signatures. Bioinformatics.

[61] Guo X, Hartemink AJ (2009). Domain-oriented edge-based alignment of protein interaction networks.. Bioinformatics.

[62] GW Klau, A new graph-based method for pairwise global network alignment. BMC Bioinformatics. 10(Suppl 1), 59 (2009). 10.1186/1471-2105-10-S1-S59 PMC2648773 19208162

[63] Memišević V, Pržulj N (2012). C-GRAAL: Common-neighbors-based global graph alignment of biological networks. Integr. Biol..

[64] M El-Kebir, J Heringa, GW Klau, *Lagrangian Relaxation Applied to Sparse Global Network Alignment vol. 7036*, (New York, 2011).

[65] Phan HTT, Sternberg MJE (2012). PINALOG: a novel approach to align protein interaction networks-implications for complex detection andfunction prediction. Bioinformatics.

[66] Chindelevitch L, Ma C-Y, Liao C-S, Berger B (2013). Optimizing a global alignment of protein interaction networks. Bioinformatics.

[67] Aladag AE, Erten C (2013). SPINAL: scalable protein interaction network alignment. Bioinformatics.

[68] Neyshabur B, Khadem A, Hashemifar S, Arab SS (2013). NETAL: a new graph-based method for global alignment of protein-protein interaction networks. Bioinformatics.

[69] Saraph V, Milenković T (2014). MAGNA: maximizing accuracy in global network alignment. Bioinformatics.

[70] Zhao H, Faisal FE, Milenkovic, T́ (2013). Global network alignment in the context of aging. Proceedings of the ACM Conference on Bioinformatics, Computational Biology and Biomedical Informatics.

[71] J Crawford, Y Sun, T Milenković, Fair evaluation of global network aligners. arXiv:1407.4824 [q-bio.MN], 17 Jul 2014 (2014). 10.1186/s13015-015-0050-8 PMC4460690 26060505

[72] J Crawford, T Milenković, GREAT: GRaphlet Edge-based network AlignmenT, arXiv:1410.5103 [q-bio.MN], 19 Oct 2014 (2014).

[73] Y Sun, J Crawford, J Tang, T Milenković, Simultaneous optimization of both node and edge conservation in network alignment via WAVE, arXiv:1410.3301 [q-bio.MN], 13 Oct 2014 (2014).

[74] SME Sahraeian, B-J Yoon, SMETANA: Accurate and scalable algorithm for probabilistic alignment of large-scale biological networks. PLOS ONE. 8(7), 67995 (2013). 10.1371/journal.pone.0067995 PMC3710069 23874484

[75] Alkan F, Erten C (2014). BEAMS: backbone extraction and merge strategy for the global many-to-many alignment of multiple PPI networks. Bioinformatics.

[76] Hu J, Kehr B, Reinert K (2014). NetCoffee: a fast and accurate global alignment approach to identify functionally conserved proteins in multiple networks. Bioinformatics.

[77] Ibragimov R, Malek M, Baumbach J, Guo J (2014). Multiple graph edit distance: simultaneous topological alignment of multiple protein-protein interaction networks with an evolutionary algorithm. Proceedings of the 2014 Conference on Genetic and Evolutionary Computation.

[78] V Gligorijević, N Malod-Dognin, N Pržulj, FUSE: multiple network alignment via data fusion. arXiv:1410.7585 [q-bio.MN], 3 Nov 2014 (2014). 10.1093/bioinformatics/btv731 26668003

[79] Ali W, Rito T, Reinert G, Sun F, Deane CM (2014). Alignment-free protein interaction network comparison. Bioinformatics.

[80] Hashemifar S, Xu J (2014). HubAlign: an accurate and efficient method for global alignment of protein-protein interaction networks. Bioinformatics.

[81] Radu A, Charleston M (2014). Node fingerprinting: an efficient heuristic for aligning biological networks. J. Comput. Biol..

[82] V Vijayan, V Saraph, T Milenković, MAGNA++: maximizing accuracy in global network alignment via both node and edge conservation. Bioinformatics (2015). doi:10.1093/bioinformatics/btv161. 10.1093/bioinformatics/btv161 25792552

[83] Ay F, Kellis M, Kahveci T (2011). SubMAP: aligning metabolic pathways with subnetwork mappings. J. Comput. Biol..

[84] Pržulj N, Corneil DG, Jurisica I (2004). Modeling interactome: scale-free or geometric?. Bioinformatics.

[85] Pržulj N, Corneil DG, Jurisica I (2006). Efficient estimation of graphlet frequency distributions in protein-protein interaction networks. Bioinformatics.

[86] ÖN Yaveroğlu, N Malod-Dognin, D Davis, Z Levnajic, V Janjic, R Karapandza, A Stojmirovic, N Pržulj, Revealing the hidden language of complex networks. Sci. Rep. 4, 4547 (2014). 10.1038/srep04547 PMC3971399 24686408

[87] Hayes W, Sun K, Pržulj N (2013). Graphlet-based measures are suitable for biological network comparison. Bioinformatics.

[88] ON Yaveroǧlu, Milenkovic, T́, N Pržulj, Proper evaluation of alignment-free network comparison methods. Bioinformatics (2015). doi:10.1093/bioinformatics/btv170. 10.1093/bioinformatics/btv170 PMC4528624 25810431

[89] T Shlomi, D Segal, E Ruppin, R Sharan, QPath: a method for querying pathways in a protein-protein interaction network. BMC Bioinformatics. 7(1), 199 (2006). 10.1186/1471-2105-7-199 PMC1458361 16606460

[90] Dost B, Shlomi T, Gupta N, Ruppin E, Bafna V, Sharan R (2007). QNet: a tool for querying protein interaction networks. Proceedings of the 11th Annual International Conference on Research in Computational Molecular Biology.

[91] G Guelsoy, B Gandhi, T Kahveci, Topac: alignment of gene regulatory networks using topology-aware coloring. J. Bioinform. Comput. Biol. 10(01) (2012). 10.1142/S021972001240001X 22809302

[92] MM Hasan, T Kahveci, in *Proceedings of the International Conference on Bioinformatics, Computational Biology and Biomedical Informatics*. Color distribution can accelerate network alignment (ACM, 2013), p. 52.

[93] Kelley BP, Sharan R, Karp RM, Sittler T, Root DE, Stockwell BR, Ideker T (2003). Conserved pathways within bacteria and yeast as revealed by global protein network alignment. Proc. Natl. Acad. Sci..

[94] Kalaev M, Bafna V, Sharan R (2008). Fast and accurate alignment of multiple protein networks. Proceedings of the 12th Annual International Conference on Research in Computational Molecular Biology.

[95] Ashburner M, Ball CA, Blake JA, Botstein D, Butler H, Cherry JM, Davis AP, Dolinski K, Dwight SS, Eppig JT, Harris MA, Hill DP, Issel-Tarver L, Kasarskis A, Lewis S, Matese JC, Richardson JE, Ringwald M, Rubin GM, Sherlock G (2000). Gene ontology: tool for the unification of biology. Nat. Genet..

[96] Boyle EI, Weng S, Gollub J, Jin H, Botstein D, Cherry JM, Sherlock G (2004). Go::termfinder-open source software for accessing gene ontology information and finding significantly enriched gene ontology terms associated with a list of genes. Bioinformatics.

[97] Brin S, Page L (1998). The anatomy of a large-scale hypertextual web search engine. Comput. Net. ISDN Syst..

[98] R Andersen, F Chung, K Lang, in *Proceedings of the 47th Annual IEEE Symposium on Foundations of Computer Science*. Local graph partitioning using pagerank vectors (Berkeley, California, USA, 2006), pp. 475–486.

[99] Pržulj N (2007). Biological network comparison using graphlet degree distribution. Bioinformatics.

[100] Milenković T, Pržulj N (2008). Uncovering biological network function via graphlet degree signatures. Cancer Inform..

[101] Milenković T, Memisević V, Ganesan AK, Pržulj N (2010). Systems-level cancer gene identification from protein interaction network topology applied to melanogenesis-related interaction networks. J. R. Soc. Interface.

[102] Solava RW, Michaels RP, Milenković T (2012). Graphlet-based edge clustering reveals pathogen-interacting proteins. Bioinformatics.

[103] GK Mazandu, NJ Mulder, Dago-fun: Tool for gene ontology-based functional analysis using term information content measures. BMC Bioinformatics. 14, 284 (2012). 10.1186/1471-2105-14-284 PMC3849277 24067102

[104] Song X, Li L, Srimani PK, Yu PS, Wang JZ (2014). Measure the semantic similarity of GO terms using aggregate information content. IEEE/ACM Trans. Comput. Biol. Bioinform..

[105] C Pesquita, D Faria, H Bastos, AO Falcão, FM Couto, in *Proceedings of the 10th Annual Bio-Ontologies Meeting*. Evaluating GO-based semantic similarity measures (Vienna, Austria, 2007), pp. 37–40.

[106] Caniza H, Romero AE, Heron S, Yang H, Devoto A, Frasca M, Mesiti M, Valentini G, Paccanaro A (2014). GOssTo: a stand-alone application and a web tool for calculating semantic similarities on the Gene Ontology. Bioinformatics.

[107] Schlicker A, Lengauer T, Albrecht M (2010). Improving disease gene prioritization using the semantic similarity of gene ontology terms. Bioinformatics.

[108] Guzzi P, Mina M, Guerra C, Cannataro M (2012). Semantic similarity analysis of protein data: assessment with biological features and issues. Brief. Bioinform..

[109] T Milenković, J Lai, N Pržulj, GraphCrunch: a tool for large network analyses. BMC Bioinformatics. 9(70) (2008). 10.1186/1471-2105-9-70 PMC2275247 18230190

[110] O Kuchaiev, A Stevanović, W Hayes, N Pržulj, GraphCrunch 2: Software tool for network modeling, alignment and clustering. BMC Bioinformatics. 12(24) (2011). 10.1186/1471-2105-12-24 PMC3036622 21244715

[111] V Janjić, R Sharan, N Pržulj, Modelling the yeast interactome. Sci. Rep. 4, 4273 (2014). 10.1038/srep04273 PMC3940977 24589662

[112] Wentz-Hunter K, Potashkin J (1995). The evolutionary conservation of the splicing apparatus between fission yeast and man. Nucleic. Acids. Symp. Ser..

[113] I Agrafioti, J Swire, J Abbott, D Huntley, S Butcher, M Stumpf, Comparative analysis of the saccharomyces cerevisiae and caenorhabditis elegans protein interaction networks. BMC Evol. Biol. 5(1) (2005). 10.1186/1471-2148-5-23 PMC1079807 15777474

[114] Forst CV, Schulten K (2001). Phylogenetic analysis of metabolic pathways. J. Mol. Evol..

[115] E Fossum, CC Friedel, SV Rajagopala, B Titz, A Baiker, T Schmidt, T Kraus, T Stellberger, C Rutenberg, S Suthram, S Bandyopadhyay, D Rose, A Brunn von, M Uhlmann, C Zeretzke, YA Dong, H Boulet, M Koegl, SM Bailer, U Koszinowski, T Ideker, P Uetz, R Zimmer, J Haas, Evolutionarily conserved herpesviral protein interaction networks. PLOS Pathogens. 5(9), 1000570 (2009). 10.1371/journal.ppat.1000570 PMC2731838 19730696

[116] Magalhães de JP, Budovsky A, Lehmann G, Costa J, Li Y, Fraifeld V, Church GMM (2009). The human ageing genomic resources: online databases and tools for biogerontologists.. Aging Cell.

[117] McGeoch DJ, Gatherer D (2005). Integrating reptilian herpesviruses into the family herpesviridae. J. Virol..

[118] McGeoch DJ, Rixon FJ, Davison AJ (2006). Topics in herpesvirus genomics and evolution. Virus Res..

